# A phantom study to optimise the automatic tube current modulation for chest CT in COVID-19

**DOI:** 10.1186/s41747-021-00218-0

**Published:** 2021-05-28

**Authors:** Victor Gombolevskiy, Sergey Morozov, Valeria Chernina, Ivan Blokhin, Jenia Vassileva

**Affiliations:** 1 Research and Practical Clinical Center for Diagnostics and Telemedicine Technologies of the Moscow Health Care Department, Moscow, Russian Federation; 2 Radiation Protection of Patients Unit, International Atomic Energy Agency, Vienna, Austria

**Keywords:** Multidetector computed tomography, Phantoms (imaging), Radiation protection, SARS-CoV-2 infection

## Abstract

On March 11, 2020, the World Health Organization declared the coronavirus disease 2019 (COVID-19) pandemic. The expert organisations recommend more cautious use of thoracic computed tomography (CT), opting for low-dose protocols. We aimed at determining a threshold value of automatic tube current modulation noise index below which there is a chance to miss an onset of ground-glass opacities (GGO) in COVID-19. A team of radiologists and medical physicists performed 25 phantom CT studies using different automatic tube current modulation settings (^SURE^Exposure3D technology). We then conducted a retrospective evaluation of the chest CT images from 22 patients with COVID-19 and calculated the density difference between the GGO and unaffected tissue. Finally, the results were matched to the phantom study results to determine the minimum noise index threshold value. The minimum density difference at the onset of COVID-19 was 252 HU (*p* < 0.001). This was found to correspond to the ^SURE^Exposure 3D noise index of 36. We established the noise index threshold of 36 for the Canon scanner without iterative reconstructions, allowing for a decrease in the dose-length product by 80%. The proposed protocol needs to be validated in a prospective study.

## Key points

- We obtained a density difference (ground-glass opacities minus visually unaffected tissue) of 252 HU (*p* < 0.001).
- The maximum diagnostic automatic tube current modulation index for ^SURE^Exposure 3D was 36.
- A dose-length product reduction by 80% was obtained, and clinical validation is needed.

## Background

On March 11, 2020, the World Health Organization declared the COVID-19 pandemic [1]. Computed tomography (CT) plays a vital role in diagnosing COVID-19, especially at the early stages. This is also true for the monitoring of disease progression and possible complications [2].

Typical manifestations of COVID-19 are ground-glass opacities (GGO) in the posterior and peripheral lung regions on CT [3, 4], often the first and only finding indicating COVID-19 [5].

The United States Center for Disease Control, the American College of Radiology (ACR), and the Royal College of Radiologists in the UK expressed caution regarding the broader use of chest CT for initial examination [6–8]. The World Health Organization published guidelines for the use of chest imaging for COVID-19, advocating the use of low-dose protocols in adults [9]. Moreover, the International Atomic Energy Agency organised a survey and a webinar to discuss CT practice and protocol optimisation for COVID-19. The resulting paper encourages using a low-dose protocol for chest CT [10]. This approach is consistent with the basic radiation protection principle of optimisation, keeping the exposure to the minimum necessary to achieve the required diagnostic objective [11].

There are currently several studies addressing the use of low-dose CT protocols in patients with known or suspected COVID-19. Kang et al. designed an imaging protocol with a volume CT dose index (CTDI_vol_) of 0.4 mGy *versus* standard-dose protocol at 3.4 mGy [12]. To achieve this, instead of the standard 120 kVp, they used lower tube voltage with a tin filter and iterative reconstruction (IR). There are no studies on developing low-dose protocols for CT scanners without IR algorithms, which are still widely available to our best knowledge.

This phantom study aimed to optimise the settings of the automatic exposure tube current modulation system of a scanner without IR algorithms and find the lowest tube current, below which scanner may cease to deliver meaningful findings in patients with COVID-19.

## Methods

The study was performed with a 64-detector CT scanner (Canon Medical Systems, Japan), equipped with a filtered back-projection (FBP) algorithm and ^SURE^Exposure 3D automatic exposure control (AEC) system. The ^SURE^Exposure 3D modulates tube current in both angular and longitudinal dimensions, based on the user-defined image quality (image noise expressed as the standard deviation [SD]) and patient’s overall attenuation [13].

The study was conducted in three phases. The first phase involved a medical physicist and a radiologist, and the second phase involved the radiologist.

### Phase I: Phantom study

We used an anthropomorphic phantom РН-1 Multipurpose Chest Phantom N1 (Kyotokagaku, Japan) measuring 43 × 40 × 48 cm, chest girth 94 cm, and adapter plates to achieve equivalency to a patient with a body mass index of 29.

A standard protocol for chest CT provided by the vendor was used. Both default and low-dose CT studies utilised the same data acquisition parameters, except for the ^SURE^Exposure 3D settings. This technology maintains automatic tube current modulation within 10–500 mA along the entire scanning region, with the 25 SD settings at the default 5.0-mm slice thickness: 10 (default), 12, 14, 16, 18, 20, 22, 24, 26, 28, 30, 32, 34, 36, 38, 40, 42, 44, 46, 48, 50, 54, 58, 62, and 68. All other scanning parameters were kept unchanged: 120 kVp, rotation time 0.5 s, direction out (craniocaudal), XY modulation on, collimation 64 × 0.5 mm, scan time 6 s, and acquired dose parameters: CTDI_vol_ in mGy and dose-length product (DLP) in mGy⋅cm.

Reconstructed images for standard and low-dose CT were identical (three reconstructions per CT scan):

1. To calibrate ^SURE^Exposure 3D: matrix 512 × 512, D-FOV 350 mm, length 300 mm, reconstruction image filter FBP QDS+, reconstruction kernel FC07 (soft tissues), thickness 5.0 mm, increment 5.0 mm, and images 60
2. Matrix 512 × 512; D-FOV 350 mm, length 300 mm, reconstruction image filter FBP QDS+, reconstruction kernel FC07 (soft tissues), thickness 1.0 mm, increment 1.0 mm, and images 300
3. Similar to number 2, except for the different reconstruction kernel FC51 (sharp kernel, lungs)

**Fig. 1:**
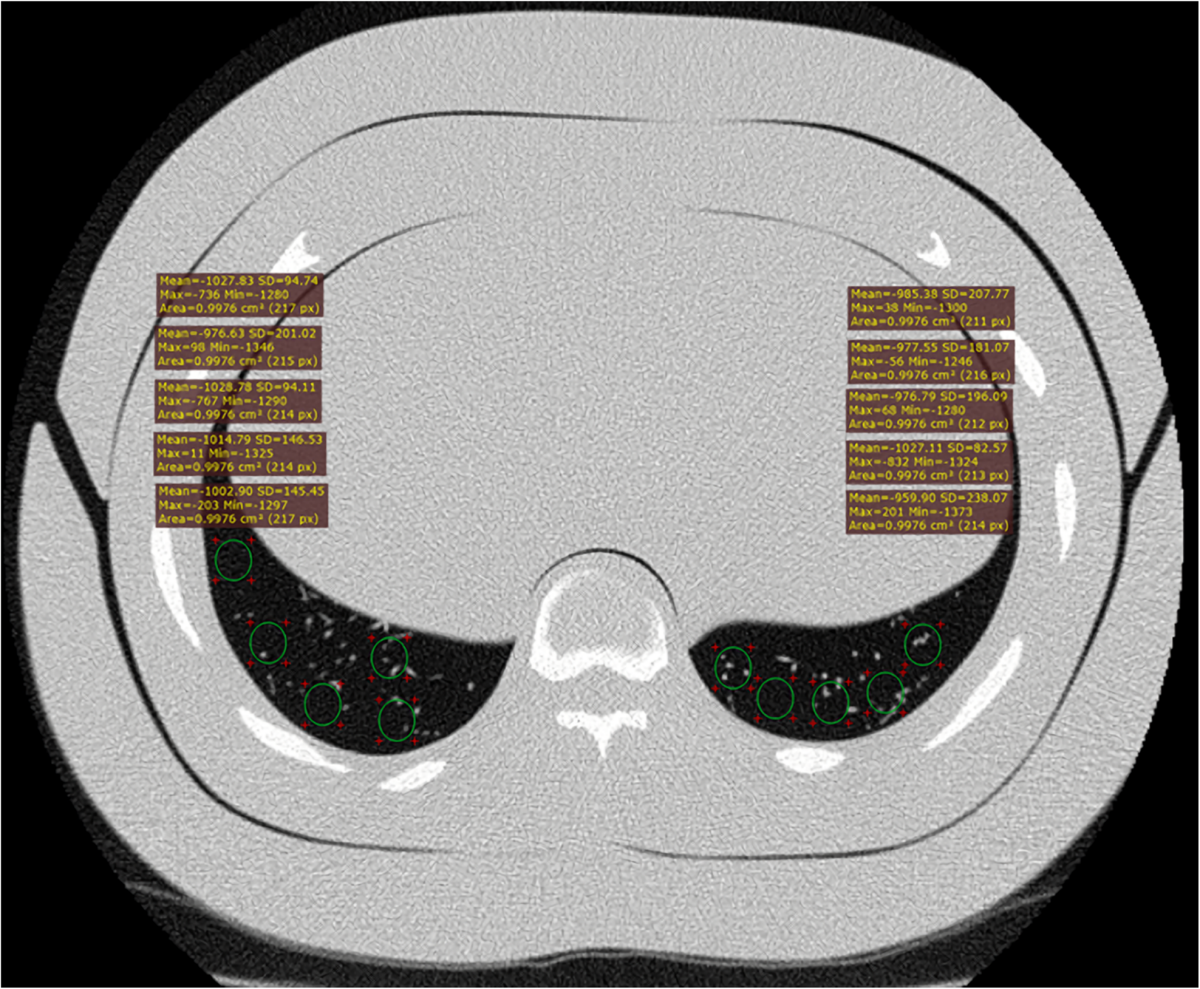
The anthropomorphic phantom РН-1 Multipurpose Chest Phantom N1 with adapter plates (an equivalent to a male patient with body mass index 29). Axial slice. Standard deviation in basal regions at Th11–12 at 1.0-mm thickness: five measurements per lung. FC51 kernel (sharp kernel, lungs). Used software: RadiAnt DICOM Viewer 5.5.1

We used the RadiAnt DICOM Viewer 5.5.1 (Medixant, Poznan, Poland), OsiriX 10.0 Lite (Pixmeo SARL, Geneva, Switzerland), and Syngo.via VB20 (SIEMENS Healthineers, Erlangen, Germany). From each scan, we measured the SD values for regions of interest (ROIs) of 1 cm^2^ at the vertebral level Th11–Th12 (Fig. 1).

The image acquisition and data analysis for the phantom study were performed by a radiologist with 10 years of experience.

### Phase II: Retrospective study

We performed a retrospective evaluation of the chest CT images obtained using the same scanner in an outpatient setting in patients with early symptoms of COVID-19. The COVID-19 was verified with a reverse-transcription polymerase chain reaction. This retrospective study, according to our regional regulations in outpatient departments, did not require ethical committee approval. We reviewed the examinations of male and female patients with age ≥ 18 years referred for chest CT by their attending physicians due to suspected community-acquired pneumonia. The inclusion criteria were as follows: a referral for chest CT with suspected pneumonia, the presence of ground-glass opacities (GGO), positive reverse-transcription polymerase chain reaction, and body mass index between 25 and 30. The exclusion criteria were as follows: age < 18 years, pregnancy or breastfeeding, implants or foreign objects in the scan area, recent chest surgery, known malignancy, and motion artifacts.

The standard CT studies were obtained with ^SURE^Exposure 3D noise index 10 for 5.0-mm image thickness. CT scans were obtained at the end of full inspiration, with patient’s arms outside the scan field, no intravenous contrast administration, and no electrocardiographic gating. CT reconstruction was performed at 1.0-mm thickness and 0.8-mm interval with kernels FC07 (soft tissues) and FC51 (sharp kernel, lungs).

The Unified Radiological Information Service (URIS) powered by AGFA HealthCare Enterprise Imaging (Agfa-Gevaert Group, Mortsel, Belgium), Syngo.via VB20 (SIEMENS Healthineers, Erlangen, Germany), and OsiriX 10.0 Lite (Pixmeo SARL, Geneva, Switzerland) were used for density measurements. ROI did not exceed 1 cm^2^ and did not include the vessels, bronchi, consolidation, and emphysema. For every patient, we measured five ROIs of GGO and five ROIs of visually unaffected tissue. The density was evaluated using 1.0-mm thickness and kernel FC51 (sharp kernel, lungs). Image analysis was performed by the radiologist with 10 years of experience.

### Phase III: Determining the ^SURE^Exposure 3D threshold

The retrospective phase provided data on the minimal difference between the average density of GGO and unaffected tissue. We matched the SD values from the retrospective phase and available phantom ^SURE^Exposure 3D data in the third phase.

### Statistical analysis

Descriptive statistics were used to report the main results; *t* test was applied to compare the densities of GGO regions and visually unaffected tissue. All the analyses were performed with the Stata14 software at the two-sided significance level of 0.05.

## Results

**Fig. 2:**
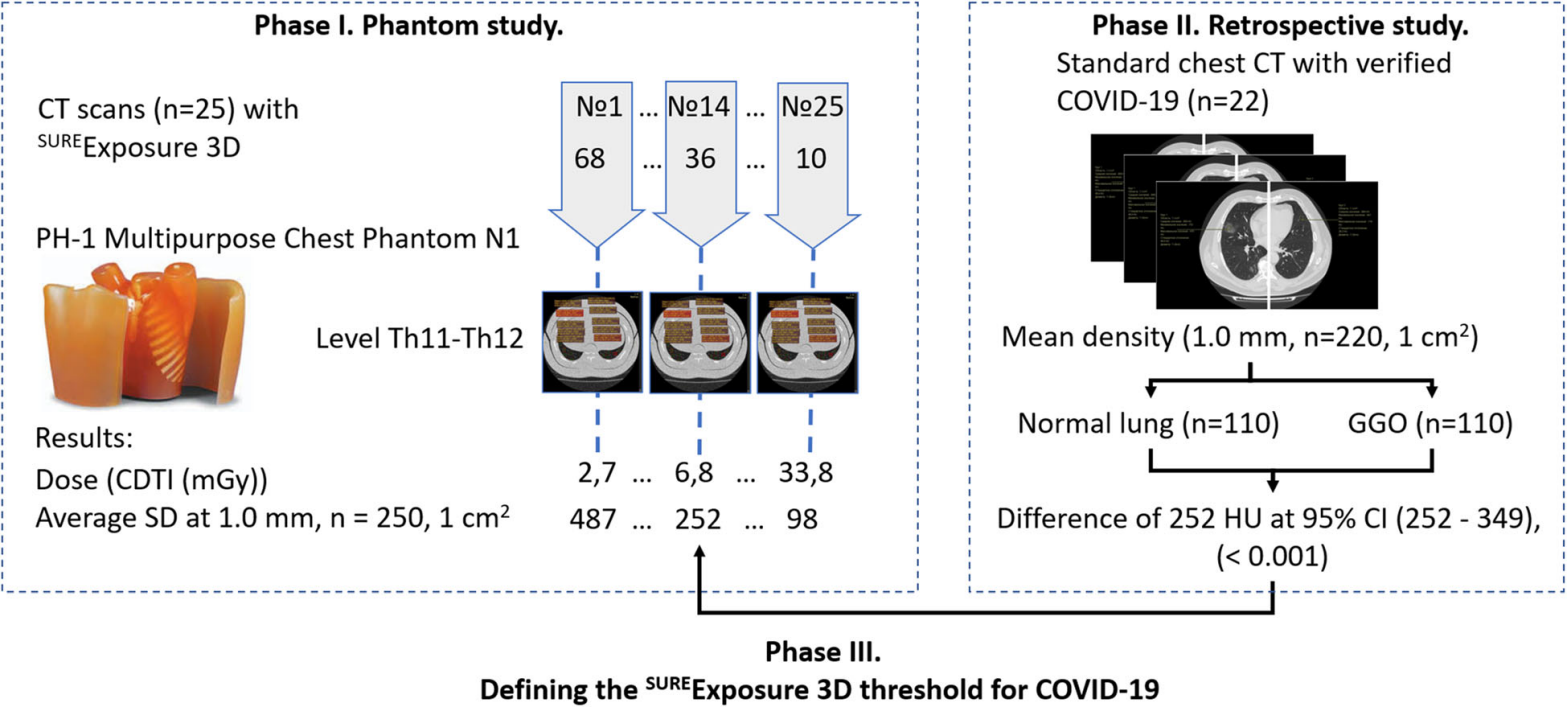
Experiment roadmap. CT, Computed tomography; SD, Standard deviation; GGO, Ground-glass opacity; CTDI, CT dose index

The roadmap and the main study parameters are summarised in Fig. 2.

**Fig. 3:**
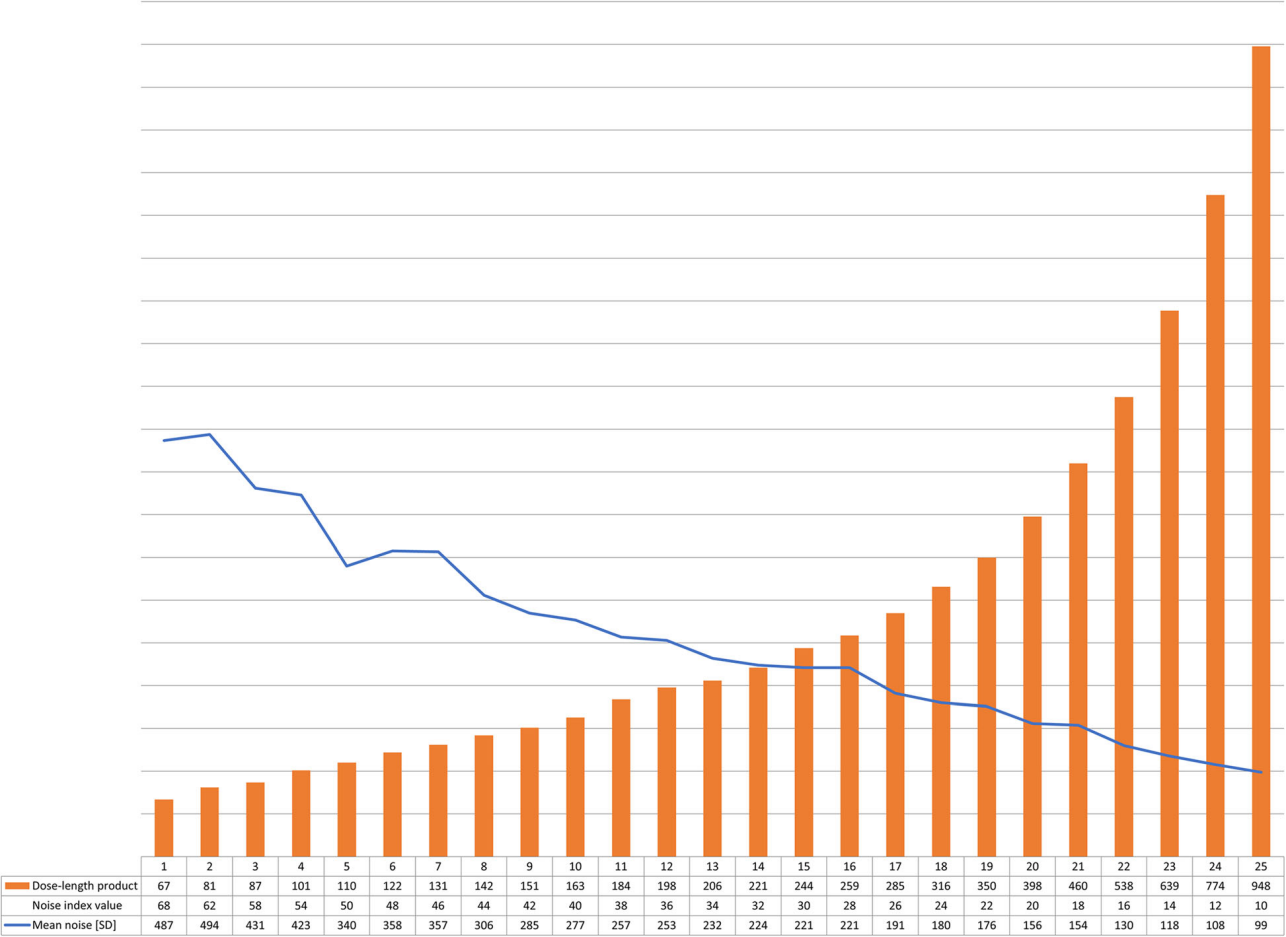
Comparison of 25 ^SURE^Exposure3D noise settings and noise standard deviation values at vertebral Th11–12 level (at 1.0-mm thickness) in the anthropomorphic phantom РН-1 Multipurpose Chest Phantom N1 (body mass index 29). Dose-length product values are in orange. The average standard deviation values are in blue

For each image, five SD measurements were performed per lung with FC51 kernel, and the average value was used for further analysis. The average SD, CTDI, and DLP values are shown in Fig. 3. A total of 250 measurements were obtained for 25 noise levels (Fig. 3).

In phase II, we analysed the clinical chest CT images of 22 patients with verified early COVID-19. The average density was assessed with ten measurements for each patient: five for GGO regions and five for visually unaffected tissue. The total number of measurements for the FC51 convolution kernel was 220. The statistical analysis of average density in HU, SD, and 95% confidence interval for the GGO and visually unaffected tissue is presented in Table 1.

**Table 1:** Density values of the ground-glass opacities and visually unaffected tissue and their difference from 22 clinical images

Group	Mean	Standard deviation	95% confidence interval	*p* value
Ground-glass opacity regions	−616.6	99.0	−662.9, −570.2	< 0.001
Visually unaffected tissue	−917.0	37.5	−934.5, −899.5	
Difference	−300.5	–	−349.2, −251.7	

Data are given as HU

The minimum difference in density was 252 HU (95% confidence interval 252–349), *p* < 0.001 (Table 1). This value was used to define the threshold noise index in phase III.

Phase III was focused on determining the ^SURE^Exposure 3D threshold (at 5.0-mm thickness), above which the difference between the GGO regions and visually unaffected tissue with FC51 kernel in patients with early COVID-19 would be imperceptible. Using the threshold of −252 HU obtained in phase II, this corresponds to a ^SURE^Exposure 3D noise index of 36 (at 5.0-mm thickness). This noise allowed decreasing radiation exposure by 80% to CTDI 6.8 mGy and DLP 198.2 mGy cm.

## Discussion

This study presents an approach to applying automatic tube current modulation at ^SURE^Exposure 3D noise index 36 (at 5.0-mm thickness) for FBP CT reconstruction, the lung kernel, and 1.0-mm thickness to determine the presence of GGO in basal lung regions at the Th11–Th12 vertebral level in patients with body mass index 29 suspected for COVID-19.

The study by Sakane et al. [14] evaluating the biological effects of low-dose chest CT demonstrated statistically significant deoxyribonucleic acid damages with standard 5.0-mSv CT compared to low-dose 1.5-mSv CT. This supports the need to develop low-dose protocols since the COVID-19 pandemic leads to more people, including the younger population, being exposed to radiation from CT scans, some of them getting repeated scans to monitor the disease progression. Simultaneously, there is a reason to believe that CT is beneficial in patients with clinically suspected or known COVID-19 in a resource-constrained environment [10].

Kalra et al. [10] demonstrated low-dose protocols for evaluating COVID-19 using flagship tomography scanners with IR from various manufacturers. Another study by Kang et al. [12] presented a COVID-19 protocol for a state-of-the-art scanner with iterative reconstruction algorithms. The protocol lowers the dose down to 0.203 mSv, which is only 1/8 of the standard protocols. However, one must keep in mind that the availability of top-tier scanners is limited in many settings. This motivated us to perform our study to find a potential for dose reduction for scanners with FBP reconstruction algorithm [15].

The histology of lesions typical for COVID-19 is very much different from those typical for other viral infections because the former comes with prominent endothelial injuries and widespread thrombosis and microangiopathy [16]. For this reason, we believe that phantom studies with tailored CT protocols can contribute to the early detection of lesions in the most affected areas. The Th11–Th12 vertebral level combines high noise level and typical lesion localisation in COVID-19 [3]. The new low-dose CT protocols should allow us to distinguish GGO even at the very onset of disease.

For this reason, it is recommended to consider the early phase of the disease when developing a new low-dose protocol [5]. Yu et al. [17] evaluated the average density of lung involvement in early COVID-19 (0–3 days) as 462 ± 99 HU. The peak density was registered between −500 and −700 HU on the density histogram. These findings are similar to the data observed in our retrospective study of patients managed in outpatient facilities. A prospective study by Schulze-Hagen et al. [18] demonstrated the accuracy of systematic low-dose chest computed tomography in the diagnosis of COVID-19 in patients with unspecific clinical symptoms with 94.7% sensitivity, 91.4% specificity, and 0.959 area under the curve. The average radiation exposure was about 1.7 mSv for a 75-kg patient.

The phantom and retrospective phases of our study utilised the 1.0-mm thickness because the ACR establishes a 1.5-mm limit for high-resolution CT [19]. Our study was not aimed towards justifying using the lung kernel, although there are certain doubts regarding whether various kernels are suitable for lung assessment. However, we decided to opt for the lung kernel for lung tissue assessment since it is recommended by the Fleischner Society [20].

This study has several limitations. First, considering the phantom phase, the findings need to be validated in a prospective clinical study. Second, clinical image reading during phase II was performed only by a single radiologist, which might have introduced bias, even though the high number of measurements could have reduced the possible bias. Third, the LungMan N1 anthropometric phantom has no way to adjust the inhale depth that affects visually unaffected tissue density.

We plan to extend this phantom study to a clinical validation in the form of a prospective transversal multireader study comparing chest CT performed with routine protocols and that performed with the proposed low-dose protocol in patients suspected to be affected with COVID-19.

In conclusion, we determined the automatic exposure control threshold for ground-glass opacity imaging in early COVID-19. A proposed low-dose protocol with a decrease in the dose-length product by 80% is promising for COVID-19 diagnostics. Further clinical studies are needed to validate the protocol.

## Abbreviations

ACR: American College of Radiology
COVID-19: Coronavirus disease 2019
CT: Computed tomography
CTDI: CT dose index
GGO: Ground-glass opacity
ROI: Region of interest

## References

[1] Listings of WHO’s response to COVID-19. https://www.who.int/news/item/29-06-2020-covidtimeline. Accessed 7 Dec 2020

[2] Xie X, Zhong Z, Zhao W, Zheng C, Wang F, Liu J (2020) Chest CT for typical coronavirus disease 2019 (COVID-19) pneumonia: relationship to negative RT-PCR testing. Radiology 296:E41–E45. 10.1148/radiol.2020200343 10.1148/radiol.2020200343 PMC7233363 32049601

[3] Li B, Li X, Wang Y, et al (2020) Diagnostic value and key features of computed tomography in coronavirus disease 2019. Emerg Microbes Infect 9:787–793. 10.1080/22221751.2020.1750307 10.1080/22221751.2020.1750307 PMC7191895 32241244

[4] Huang C, Wang Y, Li X, et al (2020) Clinical features of patients infected with 2019 novel coronavirus in Wuhan, China. Lancet 395:497–506. 10.1016/S0140-6736(20)30183-5 10.1016/S0140-6736(20)30183-5 PMC7159299 31986264

[5] Xia T, Li J, Gao J, Xu X (2020) Small solitary ground-glass nodule on CT as an initial manifestation of coronavirus disease 2019 (COVID-19) pneumonia. Korean J Radiol 21:545–549. 10.3348/kjr.2020.0240 10.3348/kjr.2020.0240 PMC7183840 32323499

[6] CDC (2020). Coronavirus Disease 2019 (COVID-19). Centers for Disease Control and Prevention.

[7] ACR Recommendations for the use of chest radiography and computed tomography (CT) for suspected COVID-19 infection https://www.acr.org/Advocacy-and-Economics/ACR-Position-Statements/Recommendations-for-Chest-Radiography-and-CT-for-Suspected-COVID19-Infection Accessed 7 Dec 2020

[8] The role of CT in patients suspected with COVID-19 infection | The Royal College of Radiologists. https://www.rcr.ac.uk/college/coronavirus-covid-19-what-rcr-doing/clinical-information/role-ct-chest/role-ct-patients Accessed 7 Dec 2020

[9] World Health Organization (2020). Use of chest imaging in COVID-19: a rapid advice guide, 11 June 2020.

[10] Kalra MK, Homayounieh F, Arru C, Holmberg O, Vassileva J (2020) Chest CT practice and protocols for COVID-19 from radiation dose management perspective. Eur Radiol 30:6554–6560. 10.1007/s00330-020-07034-x 10.1007/s00330-020-07034-x PMC7332743 32621238

[11] Radiation Protection and Safety of Radiation Sources: International Basic Safety Standards (2016). https://www.iaea.org/publications/8930/radiation-protection-and-safety-of-radiation-sources-international-basic-safety-standards. Accessed 7 Dec 2020

[12] Kang Z, Li X, Zhou S (2020) Recommendation of low-dose CT in the detection and management of COVID-2019. Eur Radiol 30:4356–4357. 10.1007/s00330-020-06809-6 10.1007/s00330-020-06809-6 PMC7088271 32193637

[13] Maldjian PD, Goldman AR (2013) Reducing radiation dose in body CT: a primer on dose metrics and key CT technical parameters. AJR Am J Roentgenol 200:741–747. 10.2214/AJR.12.9768 10.2214/AJR.12.9768 23521441

[14] Sakane H, Ishida M, Shi L, et al (2020) Biological effects of low-dose chest CT on chromosomal DNA. Radiology 295:439–445. 10.1148/radiol.2020190389 10.1148/radiol.2020190389 32154776

[15] Hong SG, Kang E-J, Park JH, et al (2018) Effect of hybrid kernel and iterative reconstruction on objective and subjective analysis of lung nodule calcification in low-dose chest CT. Korean J Radiol 19:888–896. 10.3348/kjr.2018.19.5.888 10.3348/kjr.2018.19.5.888 PMC6082754 30174478

[16] Ackermann M, Verleden SE, Kuehnel M, et al (2020) Pulmonary vascular endothelialitis, thrombosis, and angiogenesis in Covid-19. N Engl J Med 383:120–128. 10.1056/NEJMoa2015432 10.1056/NEJMoa2015432 PMC7412750 32437596

[17] Yu N, Shen C, Yu Y, Dang M, Cai S, Guo Y (2020) Lung involvement in patients with coronavirus disease-19 (COVID-19): a retrospective study based on quantitative CT findings. Chin J Acad Radiol 3:102–107. 10.1007/s42058-020-00034-2 10.1007/s42058-020-00034-2 PMC7211979 32395696

[18] Schulze-Hagen M, Hübel C, Meier-Schroers M, Yüksel C, Sander A, Sähn M, Kleines M, Isfort P, Cornelissen C, Lemmen S, Marx N, Dreher M, Brokmann J, Kopp A, Kuhl C (2020). Low-dose chest CT for the diagnosis of COVID-19—a systematic, prospective comparison with PCR. Dtsch Arztebl Int.

[19] ACR–STR practice parameter for the performance of high-resolution computed tomography (HRCT) of the lungs in adults n.d. https://www.acr.org/-/media/ACR/Files/Practice-Parameters/HRCT-Lungs.pdf Accessed 7 Dec 2020

[20] Bankier AA, MacMahon H, Goo JM, Rubin GD, Schaefer-Prokop CM, Naidich DP (2017) Recommendations for measuring pulmonary nodules at CT: a statement from the Fleischner Society. Radiology 285:584–600. 10.1148/radiol.2017162894 10.1148/radiol.2017162894 28650738

